# Adsorption of Selected Herbicides on Activated Carbon from Single- and Multi-Component Systems—Error Analysis in Isotherm Measurements

**DOI:** 10.3390/ma17174232

**Published:** 2024-08-27

**Authors:** Magdalena Blachnio, Malgorzata Zienkiewicz-Strzalka, Anna Derylo-Marczewska

**Affiliations:** Department of Physical Chemistry, Institute of Chemical Sciences, Faculty of Chemistry, Maria Curie-Sklodowska University, Maria Curie-Sklodowska Square 3, 20-031 Lublin, Poland; magdalena.blachnio@mail.umcs.pl (M.B.); malgorzata.zienkiewicz-strzalka@mail.umcs.pl (M.Z.-S.)

**Keywords:** errors in adsorption study, activated carbon, chlorophenoxyacid herbicide

## Abstract

The aim of this study is to examine the influence of various factors on the precision and repeatability of the experimental determination of herbicide adsorption isotherms. Studies were conducted for the activated carbon RIB as an adsorbent and three herbicides as adsorbates: 2,4-dichlorophenoxyacetic acid (2,4-D), 4-chlorophenoxyacetic acid (4-CPA), and 3-chlorophenoxypropionic acid (3-CPP). The herbicide adsorption process was carried out in single-component and multi-component modes (the herbicide was adsorbed in the presence of an accompanying substance, i.e., 4-nitroaniline (4-NA)). Due to the significant contribution of the competition phenomenon in the adsorption process, which is important, e.g., in multi-component environmental systems, a qualitative and quantitative analysis of herbicide adsorption in the presence of a competing substance was presented. This work presents, among other things, the influence of adsorbent heterogeneity (grain size) on measurement uncertainties. The spread of standard deviations for solutions requiring dilution during spectrophotometric measurements was discussed, indicating that dilutions contribute to increasing measurement uncertainties. The heterogeneity parameters of the Freundlich equation for the studied adsorption systems were analyzed; the 2,4-D/RIB system was indicated as the most energetically heterogeneous. Differentiation of the experimental conditions (pH, temperature) allowed us to assess their impact on the efficiency and mechanism of adsorption. A high repeatability of experimental isotherms was obtained for the multi-component system. The accuracy of quantitative determination of equilibrium concentrations for the tested two-component systems was assessed based on the measured UV-Vis spectra, and the adsorption of herbicides from single- and multi-component systems was compared.

## 1. Introduction

Valuable scientific studies and literature reports prove the great importance of the issue of errors and uncertainty in adsorption experiments [1,2,3,4,5,6]. Statistics plays a crucial role in adsorption research, providing essential tools for experimental design, data analysis, and model fitting and hypothesis testing [7]. At the stage of designing and planning an adsorption experiment, statistical data can be useful for assessing the effects of multiple factors (e.g., temperature, pressure, adsorbent dosage) on adsorption efficiency [8]. This has an impact on the optimizing conditions with minimal experimental effort [9]. Statistical analysis of experimental data enables the summarization of key metrics such as mean, median, mode, standard deviation, and variance, assisting understanding of the central tendency and variability in adsorption data. Additionally, it facilitates correlation analysis, which examines the relationships between different variables (e.g., temperature and adsorption capacity) to identify the key factors that influence adsorption phenomena. Statistical tools are employed in fitting procedures by using adsorption isotherms (e.g., Langmuir [10,11], Freundlich [12], Temkin [13], and also a new modeling approach that gives satisfactory fitting results [14]), enabling the determination of parameters like adsorption capacity and affinity. The validation assessment criteria may be linear regression coefficient (R^2^), the sum of the squares of the errors (SSE), the hybrid fractional error function (HYBRID), the average relative error deviation (ARED) and the residual analysis (RESID), or others [15,16,17,18]. The validity of the experimental data is also crucial for fitting adsorption kinetic data to suitable models (e.g., pseudo-first-order, pseudo-second-order), which helps in understanding the rate-controlling steps and predicting adsorption behavior over time [19,20,21]. Statistical analysis in adsorption studies also helps in understanding interactions between variables and optimizing the adsorption process by fitting theoretical models. It enables the determination of the significance of observed effects (e.g., the impact of pH on adsorption) and the validation of experimental findings [22]. Additionally, it facilitates the scaling up of adsorption processes from the laboratory to industrial scale by correlating laboratory data with process conditions. Adsorption studies of environmentally important systems are a substantial element of currently developed research directions [23,24,25,26]. On one hand, there is a focus on utilizing modern, natural, and multifunctional materials as adsorbents [27,28,29]; on the other hand, the type of substances being removed remains a crucial consideration. Substances that are particularly interesting due to their high level of practical use and health consequences for humans and living organisms include herbicides and their derivatives [30,31,32,33,34]. Scientific research in this area confirms that sometimes even a small change in the process conditions, and physicochemical properties of the adsorbent, including its form, selection of reagents, and external factors, may be responsible for changing the efficiency of the entire process [35,36,37]. At this point, the aspect of measurement precision and potential experimental errors, which may also result in a similar effect, should also be evaluated [38,39,40,41]. Errors in the adsorption tests may result from various sources, either in the sample preparation phase, conducting the experiment, and data analysis [6,42]. Understanding and minimizing possible errors is key to obtaining accurate and repeatable results in adsorption studies. Regular calibration of equipment, control of experimental conditions, and the use of appropriate data analysis models can significantly improve the reliability of the results obtained.

Considering the type of factors influencing the measurement result of adsorption isotherms of organic pollutants, possible errors can be divided into random errors, systematic errors, and gross errors. Random errors are characterized by their varying values in subsequent measurements conducted in the same manner. In other words, random errors occur when repeating a measurement experiment under an unchanging set of physical conditions, and reveal random variability in the results. Random errors are caused by the influence of many variables and usually independent factors. These errors may arise from (i) changes in the molecular form of the adsorbate; (ii) the heterogeneity of adsorbent samples; (iii) uneven degassing of the adsorbent; (iv) inaccurate measurement of adsorbate solutions; (v) non-quantitative introduction of solutions into flasks; (vi) the variability of external factors; (vii) the inaccurate dilution of post-adsorption solutions; or (viii) the instability of the analytical balance and spectrophotometer device. Systematic errors result from consistent influencing factors. They have a constant value in repeated measurements under the same conditions, typically causing a shift in the same direction. Possible causes include (i) incorrect instrument settings; (ii) insufficient chemical purity of the adsorbate; (iii) external disturbances to the measurement system; or (iv) imperfect standardization or calibration. Gross errors are due to mistakes during experimental setup, measurement, or data recording, such as (i) misplacement of the decimal point when recording the mass of substances; (ii) faulty operation of instruments; (iii) inaccurate reading of absorbance values from spectroscopic spectra; or (iv) calculation errors when preparing graphs. Gross errors are usually easy to notice and eliminate because the obtained result differs significantly from other measurement results of the same physicochemical quantity. Although the number of variable parameters affecting the quality of experimental research is considerable, research conducted in this area remains valuable and helps to improve the efficiency of adsorption processes. Examples include works in which the effects of various operating parameters and error analysis were evaluated for adsorption isotherm models for the adsorption of penicillin G onto magnesium oxide nanoparticles [43], acid dyes onto activated biocarbon [39], phenol onto coconut shell-based activated carbon [44], metal ions onto leonardite [45], p-nitrophenol onto activated carbon [46], and many others [47,48].

Considering the widespread use of adsorption processes, a very important issue, in addition to developing new types of adsorbents, is the reliability of experimental measurements. Due to the time-consuming nature of experimental measurements of adsorption isotherms, there are only a few publications available in the literature presenting an analysis of measurement errors, taking into account the influence of many factors on the obtained results. In this work, the effect of various factors on the precision and repeatability of the adsorption of selected organic pollutants on the activated carbon RIB using a spectrophotometric method was studied. The adsorption measurements were carried out in single- and multi-component modes in four individual series, repeating the entire experimental cycle each time. For the determination of the equilibrium concentration of solutions for binary systems, the law of additivity was used. Based on the standard deviations for absorbance and equilibrium concentrations, assessments of measurement errors of the experimental points on the adsorption isotherms were made. For an analysis of adsorption data in single-component systems, the Freundlich model was applied and the values of parameters m and log K and their uncertainties along with the weighted means were determined. For each measurement series of the adsorption system, the values of uncertainty for the adsorption logarithm and correlation coefficient were also obtained. The obtained results indicated, first of all, the significant role of the non-homogeneity of the adsorbents on the random errors of adsorption isotherm measurements. Differentiations in adsorbent grain sizes and structural and energetic heterogeneity result in deviations observed in the experimental data measured for the series of adsorption systems. The influence of other factors and measurement methodologies was also discussed.

## 2. Materials and Methods

### 2.1. Adsorbent and Adsorbates

The activated carbon RIB (Norit NV, Amersfoort, The Netherlands) belonging to the series of activated carbons formed (cylinders) was used as an adsorbent. Prior to the experiment, the carbonaceous material was treated with hydrochloric acid at 60 °C for 6 h to remove mineral impurities (ash). The herbicides 2,4-dichlorophenoxyacetic acid (2,4-D); 4-chlorophenoxyacetic acid (4-CPA) (Sigma-Aldrich, St. Louis, MO, USA); and 3-chlorophenoxypropionic acid (3-CPP) (synthesized and purified in the Department of Organic Chemistry at Maria Curie-Sklodowska University, Gliniana Street, Lublin, 20-614, Poland) were used as adsorbates. Additionally, 4-nitroaniline (4-NA) (Sigma-Aldrich, St. Louis, MO, USA) was selected as an accompanying substance in a multi-component system. The physicochemical properties of the studied adsorbates are listed in Table 1.

### 2.2. Methods and Techniques of Activated Carbon Characterization

The porous structure of the activated carbon RIB was evaluated using a nitrogen adsorption/desorption isotherm at 77 K, spanning a relative pressure range of 0 to 950 mmHg, with an ASAP 2020 analyzer (Micromeritics, Norcross, GA, USA). The specific surface area (S_BET_) was calculated from the experimental isotherm following the standard Brunauer–Emmett–Teller (BET) method. Pore size distribution curves were obtained from the adsorption and desorption branches of the isotherm using the Barrett–Joyner–Halenda (BJH) model for cylindrical pores and the Faas correction, as well as the Horvath–Kawazoe (HK) method (slit geometry) and non-local density functional theory (NLDFT) assuming slit geometry for the carbon porosity model. The total pore volume (V_t_) was determined from the amount of adsorbed nitrogen at p/p_0_ = 0.97, while the micropore volume (V_mic_) was estimated using the t-plot method. RIB carbon was degassed at 100 °C and 1 mmHg for 24 h in the analyzer’s degassing port before analysis. The acid–base features of the carbon surface were determined through potentiometric titration measurement. An acidified suspension of the carbon (0.1 g of carbon), using NaCl as the diluent (30 mL), was placed in a thermostatic vessel at 25 °C and titrated with a NaOH solution using an automatic burette (Dosimat 765, Metrohm, Herisau, Switzerland) connected to a pH meter (PHM240, Radiometer, Copenhagen, Denmark). From the pH changes as a function of titrant volume, the surface charge density and point of zero charge value for the activated carbon were determined. The solid surface topography was examined using transmission electron microscopy (Tecnai G2 T20 X-TWIN, FEI Company, Hillsboro, Oregon, United States). The chemical characterization of the surface chemistry of the adsorbent, which provides information about the surface-active groups, the elemental composition, and the electronic state of the elements, was carried out using X-ray photoelectron spectroscopy (XPS). X-ray photoelectron spectroscopy data were collected on a Multi-chamber UHV System, Prevac (2009, Rogów, Poland) using the hemispherical analyzer ScientaR4000 by monochromatic Al Ka radiation from a high-intensity source MX-650, Scienta (Uppsala, Sweden).

### 2.3. Measurement of Adsorption Isotherms from Aqueous Solutions

The adsorption isotherms of organic substances from single- and multi-component systems on activated carbon were measured using the static method. In this experiment, 0.05 g samples of the activated carbon RIB were placed with 5 mL of distilled water (or specific pH electrolyte) in Erlenmeyer flasks and degassed under a vacuum. Then, 100 mL herbicide and/or accompanying substance solutions of defined concentrations (2,4-D: 0.43–2.15 mmol/L; 4-CPA: 0.51–2.56 mmol/L; 3-CPP: 0.34–3.03 mmol/L; 4-NA: 0.09–3.12 mmol/L; pollutant mixture: 0.133–3.323 mmol/L; and 0.066–1.654 mmol/L for 3-CPP and 4-NA, respectively) were added to the flasks. The prepared adsorption systems were shaken in an Innova 40 incubator at a speed of 110 rpm and at the constant temperature of 25 °C for 10 days. For 2,4-D and 4-CPA, the experiments were also conducted at 45 °C. Adsorption from a multi-component system was only performed for 3-chlorophenoxypropionic acid (3-CPP) in the presence of 4-nitroaniline (4-NA). The molar ratio of the pesticide to the accompanying substance in the initial solutions was 2 to 1. The adsorption experiments for 2,4-D and 4-CPA were conducted under acidic conditions (pH = 2), and under neutral conditions (pH~7) for the others (3-CPP, 4-NA, 3-CPP + 4-NA). After reaching adsorption equilibrium, the absorption spectra of the solution samples were measured using the spectrophotometer Cary 4000 (Varian Inc., Palo Alto, CA, USA ). Measurements of adsorption isotherms from single and binary aqueous solutions were performed for four series, repeating the entire experimental cycle each time.

## 3. Results and Discussion

### 3.1. Adsorbent Characterization

**Figure 1:**
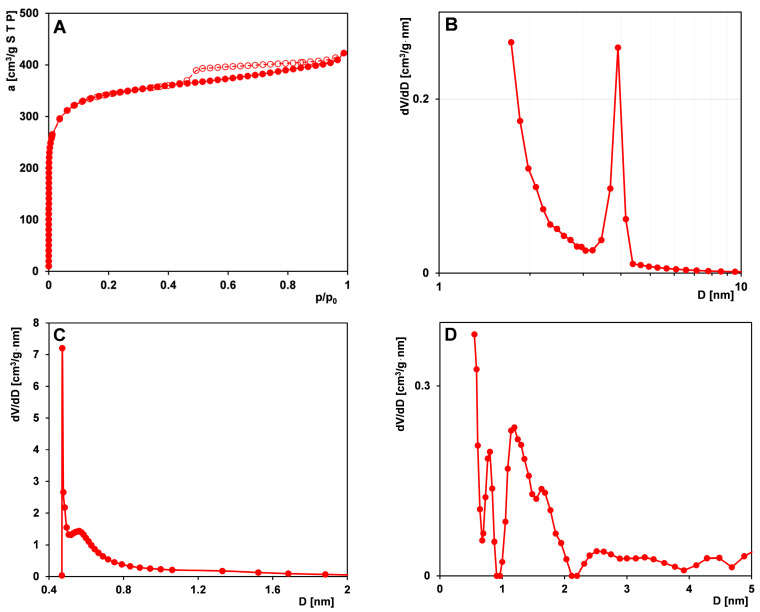
(**A**) Nitrogen adsorption–desorption isotherm for the activated carbon RIB, (**B**) BJH pore size distribution curve from desorption branch, and (**C**) micropore size distributions determined from Horvath–Kawazoe (HK) and (**D**) non-local density functional theory (NLDFT).

Figure 1A shows the nitrogen adsorption–desorption isotherm for the carbon material. The isotherm is classified as type I with a hysteresis loop H4 according to the International Union of Pure and Applied Chemistry (IUPAC) classification. The shape of the experimental curves suggests a great amount of micropores (acute initial course) and also a certain amount of mesopores. A capillary condensation within the mesopores occurs at a relative pressure from 0.45 to 0.8 (p/p_0_) and confirms the substantial presence of narrow and slit mesopores. The BET-specific surface area (S_BET_) and total pore volume (V_t_) of investigated carbon equal 1052 m^2^/g and 0.65 cm^3^/g, respectively (Table 2). The BJH pore size distribution study (Figure 1B) indicates that the carbon material has the largest fraction of mesopores with diameters~3.9 nm. Based on the pore size distribution calculated from the adsorption isotherm using the HK model [53], the corresponding average micropore size D_mo_ (HK) of RIB is 0.66 nm with a distribution range of sizes from 0.45 nm to 0.8 nm (Figure 1C). The NLDFT data clarify the size distribution of micropores (Figure 1D).

**Figure 2:**
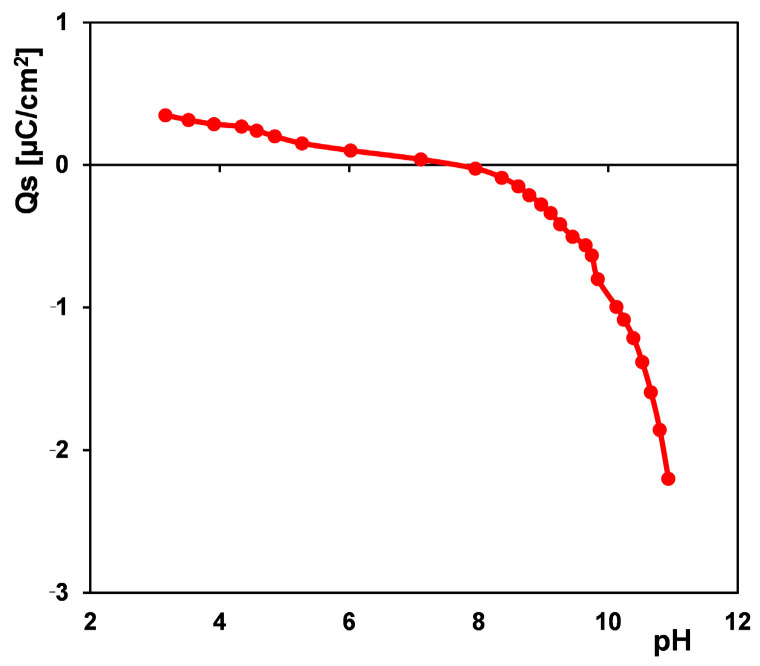
Dependence of surface charge density vs. solution pH for the activated carbon RIB.

Figure 2 shows the dependence of the surface charge density of a solid on the solution pH. Based on this dependence, one can determine the zero point value, i.e., the pH of the solution for which the charge of the solid is zero (pH_pzc_), and indicate the net surface charge of the carbon in the given experimental conditions. Generally, the acidity/basicity of activated carbons is determined by the presence of various surface functional groups on their surface and the pH of the solution. The acidic properties are due to oxygen-containing species such as carboxylic, lactonic, lactol, and phenolic. In turn, the basic properties of materials are ascribed to chromenic, pyronic, or quinone groups, and the π-electron system of carbon basal planes [54]. The qualitative and quantitative content of surface moieties depends on the activated carbon synthesis method and the nature of the precursor [55,56]. When the charges from both types of functional groups (acidic and basic) are balanced, the total surface charge of the solid is zero (pH_pzc_). The point of pH at which the activated carbon RIB has zero charge is 7.8, so at a solution pH lower than this value, its surface charge is positive (the protonation of the surface moieties plus the carbon graphene layers acting as Lewis bases), while at a higher solution pH, its surface charge is negative (the ionization of acidic oxygen surface groups). The experimental conditions of the adsorption process were pH = 2 or pH~7, which suggests the maintaining of a positive charge by the solid. Under acidic conditions, the surface charge of the adsorbent was higher than under neutral because a slight part of the acid functional groups was ionized.

**Figure 3:**
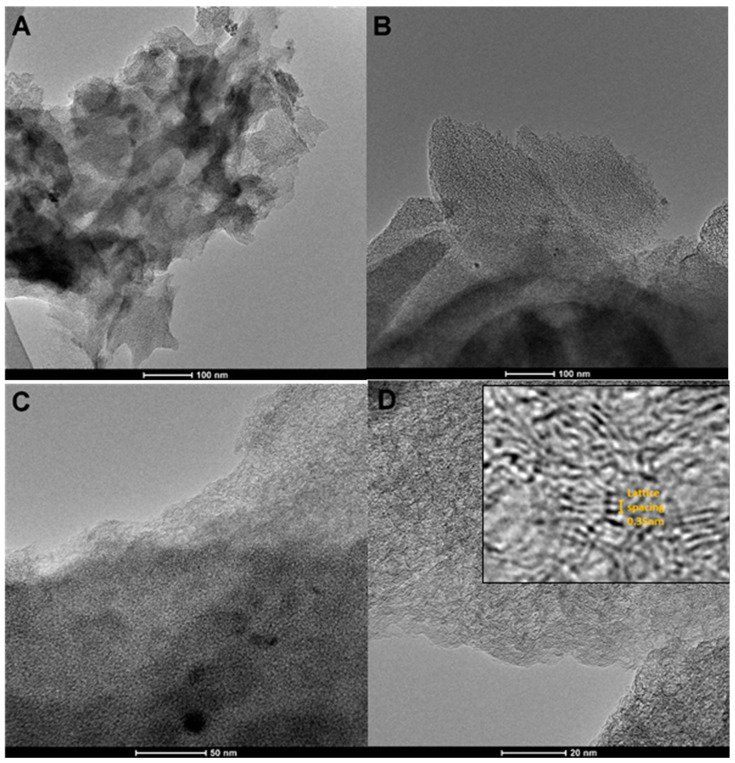
(**A**–**D**) Transmission electron micrographs (TEMs) for the activated carbon RIB at various magnifications. A magnification of the selected area of the TEM image (**D**) is shown as an inset of this image.

The microstructure of the activated carbon RIB is presented in Figure 3. The carbonaceous material displays a multi-dimensional, non-uniform structure with carbon planes spaced at a distance of approximately 0.35 Å (inset in Figure 3D). Carbon planes appear distinctly parallel to the electron beam and create slit-porous spaces for adsorption phenomena. As can be seen in several TEM photos, such a microstructure is repeatable throughout the entire volume of the carbon sample.

**Figure 4:**
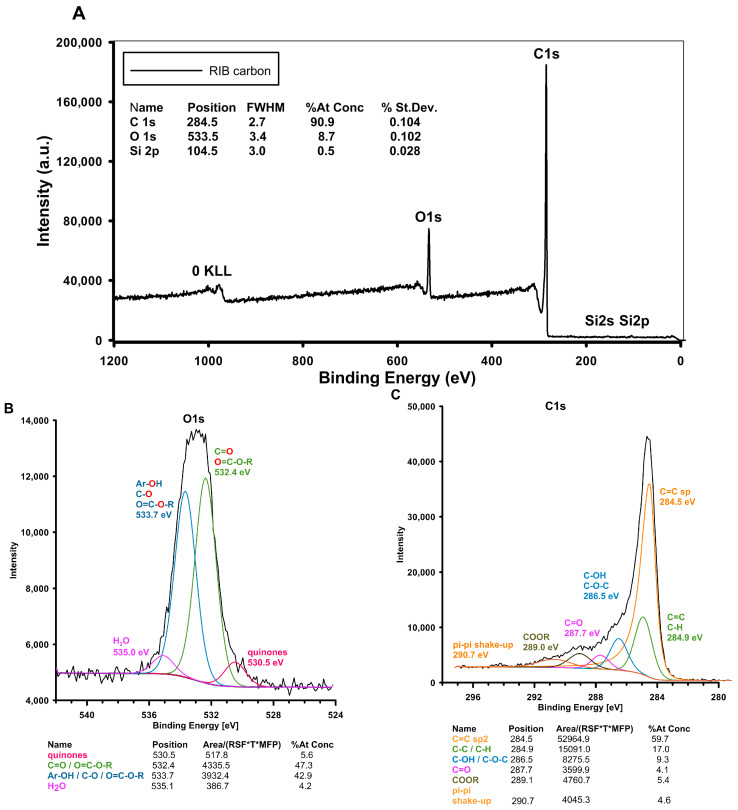
(**A**) Survey scan XPS spectra of the carbon RIB and details of elemental analysis. (**B**) High-resolution core-level spectra from the O1s region and (**C**) high-resolution core-level spectra from the C1s region with identification and contents of individual core levels.

X-ray photoelectron spectroscopy (XPS), a powerful analytical technique, was used to study the surface chemistry of the investigated RIB carbon. The primary aim of XPS is to determine the elemental composition, chemical state, and electronic state of the elements present on the surface of a material. The obtained results identified the elements present on the surface of carbon by detecting the characteristic binding energies of electrons ejected. Figure 4A shows the survey scan and high-resolution spectra of the RIB carbon surfaces. The details of the XPS analysis (atomic concentration and position of each element) are also presented in Figure 4. The XPS spectra of RIB carbon indicate the presence of carbon, oxygen, and trace amounts of silicon. The main peaks of O1s atomic level at the positions of 532.4 eV and 533.7 eV are attributed to the C=O or O=C-O-R and -O-C- bonding, respectively. The proportions of the -C=O and -C-O- groups were 47.3% and 42.9% and constitute the majority of oxygen species on the surface of the carbon material. This indicates the presence of acidic forms on the surface of the analyzed carbon, which may occur in a dissociated or undissociated form under the experimental conditions and have a repulsive effect on the anionic form of the 3CPP adsorbate (dissociated form of acid groups and lower adsorption of this type of herbicide). Moreover, the highly conjugated forms of carbonyl oxygen species such as quinone groups were identified at 530.5 eV. Figure 4C shows several carbon-containing species. The main peak of the sp3 carbon of C-C in the graphitic structure was confirmed at 284.5 eV. The proportional content of these forms was 59.7%. Next, the higher binding energy peak at 284.9 eV was ascribed to the alkyl-type carbon form (content ~17%). Moreover, the C1s spectrum includes three peaks attributed to the oxygen-containing functional groups. Peaks at 286.5 eV, 287.7 eV, and 289.1 are attributed to the carbon atoms in the C-OH/C-O-C, C=O, and COCR functional groups, respectively. In the C1s spectra, the band at 290.7 eV was attributed to the shakeup peak associated with a π–π transition. The presence of the latter forms is important due to the participation of π electrons in the adsorption mechanism of 2,4-D and 4-CPA adsorbates described in the section devoted to this process in the further parts of the paper.

### 3.2. Error Analysis in Measurements of Adsorption Isotherms from Single-Component System

**Figure 5:**
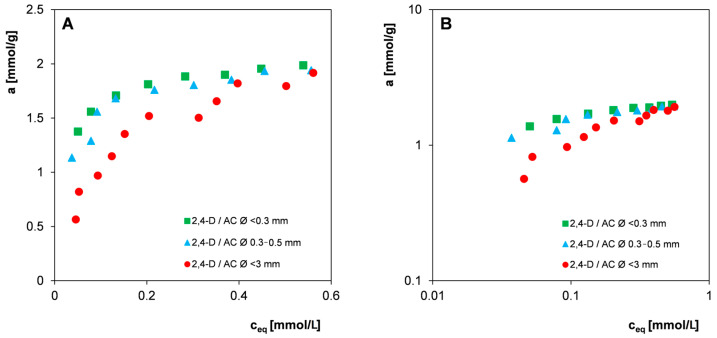
Comparison of the 2,4-D adsorption isotherms on exemplary activated carbon (AC) with different grain fractions (different sizes) applied in the linear (**A**) and logarithmic coordinates (**B**).

In the first stage of the analysis of potential errors, the impact of the grain size of the carbon material was considered (Figure 5). To illustrate the influence of grain size on the diversity of the results obtained, the carbon adsorbent (exemplary activated carbon AC, not RIB) was sieved to obtain two fractions with grain sizes of <0.3 mm and 0.3–0.5 mm. For comparison, both of these fractions and also unscreened carbon (grain size < 3 mm) were used for the measurement of the adsorption isotherms of the 2,4-D herbicide.

The large scatter of experimental points for individual activated carbon fractions confirms the significant impact of adsorbent heterogeneity on measurement uncertainties. In the case of two fractions with a narrow grain distribution (<0.3 mm; 0.3–0.5 mm), the scatter of experimental points is much smaller than for those containing granules of different sizes from the range < 0.3–3 mm. Moreover, quite significant differences in the course of the 2,4-D adsorption isotherms should be noted, especially in the area of lower concentrations for the three tested fractions. At the same equilibrium concentration values, stronger adsorption is observed on carbon with smaller grains of similar size compared to the unscreened sample containing both small and large grains. This effect should be associated with the slower diffusion of adsorbate molecules in the internal space of large granules and the resulting slower achievement of the equilibrium state. Due to the reduced availability of the part of the adsorbent in which the process occurs quickly, the adsorbent with large grains in a time-limited adsorption experiment behaves as if it had a lower adsorption capacity and a lower adsorption equilibrium constant.

**Figure 6:**
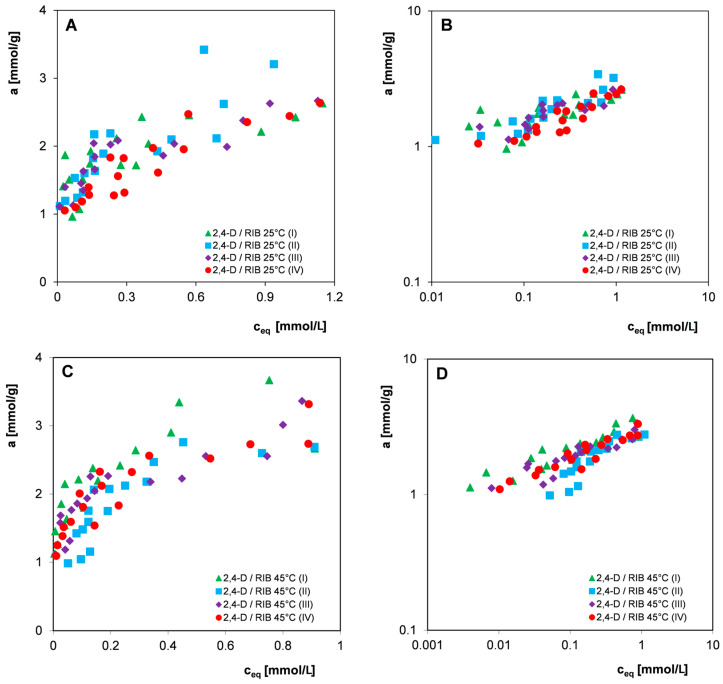
Comparison of adsorption isotherms of the herbicide 2,4-D at 25 °C (**A**,**B**) and 45 °C (**C**,**D**) on the activated carbon RIB measured in four series in linear (**A**,**C**) and logarithmic coordinates (**B**,**D**).

**Figure 7:**
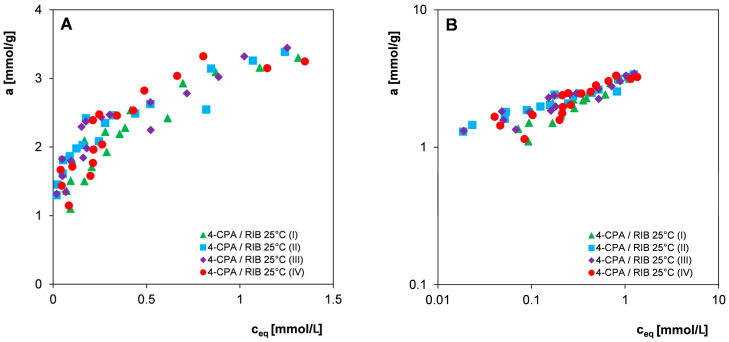

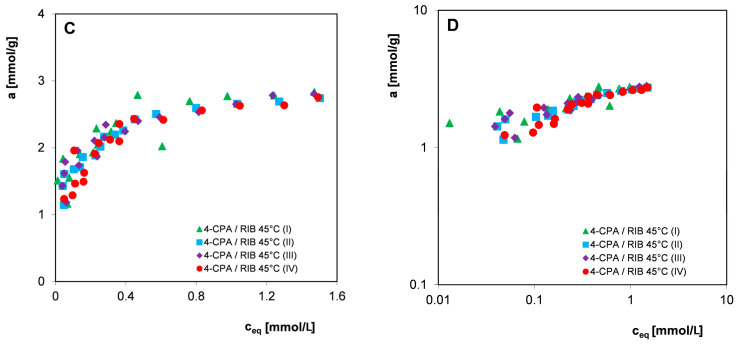
Comparison of adsorption isotherms of the herbicide 4-CPA at 25 °C (**A**,**B**) and 45 °C (**C**,**D**) on the activated carbon RIB measured in four series in linear (**A**,**C**) and logarithmic coordinates (**B**,**D**).

**Figure 8:**
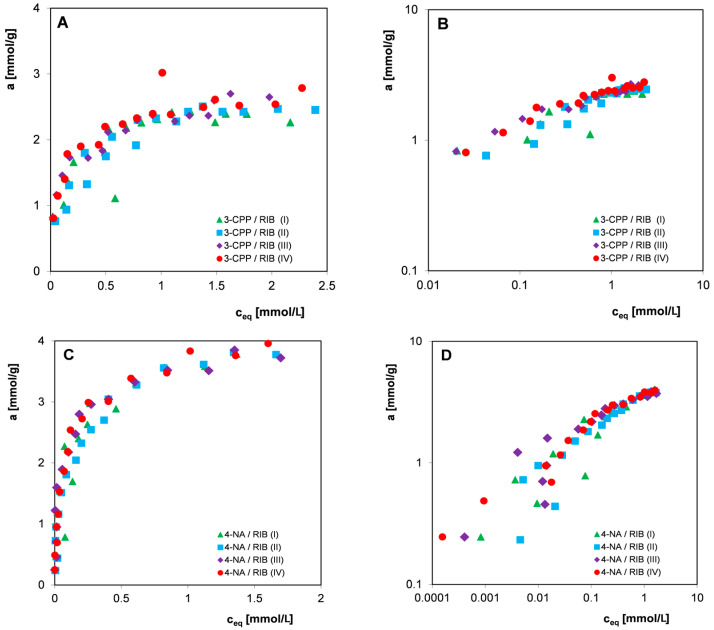
Comparison of adsorption isotherms of the herbicide 3-CPP (**A**,**B**) and 4-NA (**C**,**D**) on the activated carbon RIB measured in four series in linear (**A**,**C**) and logarithmic coordinates (**B**,**D**).

Since in most practical applications, commercial materials with a fairly large grain size distribution are used, all further studies on the influence of adsorbent heterogeneity and the potential influence of other factors on the measurement uncertainties of the organic compounds adsorption were carried out without preliminary sifting of adsorbent. Qualitative and quantitative analysis was performed for three selected 2,4-D herbicides: 4-CPA and 3-CPP and the nitro derivative 4-NA in systems with the carbon RIB. The experimental points constituting the adsorption isotherms were obtained from measurements carried out from aqueous solutions of electrolytes with pH = 2 at temperatures of 25 °C and 45 °C (2,4-D and 4-CPA) or from water at a temperature of 25 °C (3-CPP and 4-NA) by repeating the procedure of preparing and performing the measurement series four times. Figure 6, Figure 7 and Figure 8 show the adsorption isotherms for individual adsorbates in linear and logarithmic coordinates. Such a presentation of the experimental data allows us to compare the adsorption isotherms in higher (linear) and lower (logarithmic) concentration ranges.

Analyzing the presented relationships (Figure 6, Figure 7 and Figure 8), a certain scatter of experimental points can be observed both in the areas of high and low concentrations (visible in the logarithmic graph), although in the case of all pollutants considered, the spread is higher in the area of lower equilibrium concentrations. In the case of 4-NA (Figure 8C,D), the smallest scatter of experimental points was observed, the individual isotherms of four measurement series were very similar, and some of the experimental points even overlapped. For 2,4-D, the largest scatter of points was observed. Because the measurement series were performed according to strictly established procedures for all organic compounds, differences in the size of the scatter of experimental points can be attributed to their different physicochemical properties, such as changes in molecular form as a function of solution pH or the degree of purity of adsorbates. However, no significant influence of the temperature conditions of the adsorption process on measurement uncertainties was observed.

The results of adsorption measurements presented in this study do not significantly differ from others, so the absence of gross errors was assumed. In the case of random and systematic errors, the method of measuring equilibrium isotherms influences which type of errors may prevail. Generally, there are two methods for measuring equilibrium isotherms: (i) using many independent portions of the adsorbent and adsorbate solutions and (ii) using one portion of the adsorbent and subsequent repeated dosing of the adsorbate. In the first method and the one used in our experiment, it should be expected that the results for each isotherm measurement point are subject to random errors related to the non-homogeneity of the adsorbent sample (differentiation of grain sizes, and the physicochemical and structural properties of individual granules included in a single sample). On the other hand, the chosen method allows us to avoid systematic errors related to taking a small sample of the adsorbent (the uncertainty is inversely proportional to the square root of the mass of the sample) and imprecise determination of the adsorbate concentration before introducing its next portion into the adsorption system.

A measurement error of the experimental point “*j*” on the adsorption isotherms was assessed based on the determined values of *SD_j_* (standard deviation) for absorbance (*SD_A_*) (Equation (1)) and equilibrium concentrations (*SD_c eq_*) (Equation (2)):

(1) $${SD}_{A(j)}={\left[\sum_{i=1}^{L}{\left(A_{i\left(j\right)exp}-A_{\left(j\right)av}\right)}^{2}/\left(L-1\right)\right]}^{1/2}$$

(2) $${SD}_{c\;eq(j)}={\left[\sum_{i=1}^{L}{\left(c_{eqi\left(j\right)exp}-c_{eq\left(j\right)av}\right)}^{2}/\left(L-1\right)\right]}^{1/2}$$

where *A_i(j)exp_*—absorbance of the point “*j*” determined experimentally, *A_(j)av_*—average absorbance of the point ”*j*” from four measurement series, *L* = 4—number of measurement series, *c_eq i(j)exp_*—equilibrium concentration of the point “*j*” determined experimentally, *c_(j)av_*—average concentration of the point “*j*” calculated from four measurement series.

**Figure 9:**
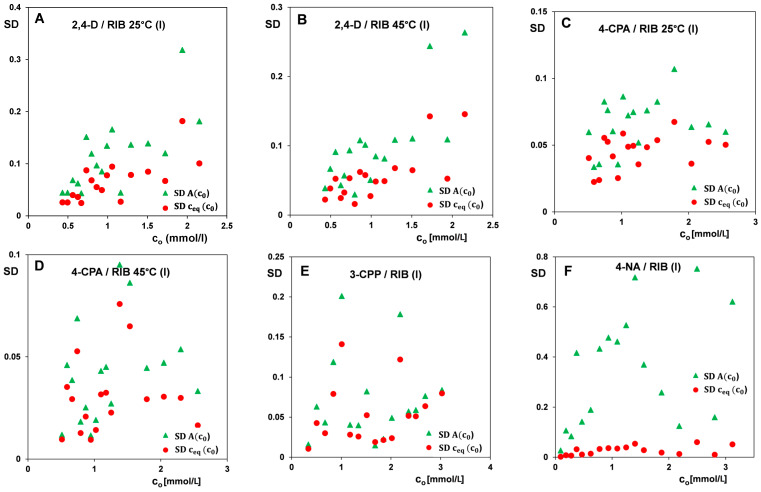
Correlations of standard deviations for determination of equilibrium concentration and absorbance on initial concentration for the selected series of adsorption measurements of 2,4-D at 25 °C and 45 °C (**A**,**B**), 4-CPA at 25 °C and 45 °C (**C**,**D**), 3-CPP (**E**), and 4-NA (**F**).

The correlations of standard deviations for the determination of the equilibrium concentration and absorbance as a function of the initial concentration for the selected measurement series obtained for the tested adsorption systems are presented in Figure 9. By analyzing the measurement uncertainties, it was found that they approximately increased with the measured value (this trend was the strongest in the case of 2,4-D/RIB, but much weaker for other systems).

Due to the adsorption range (min/max), quite a large spread of points, and their relatively small number (incomplete statistics—usually quite weak correlations), it can be practically equivalently assumed that such a dependence is only partial, i.e., without making a large error, it can also be assumed that there is no such dependence (uncertainty independent of the amount of adsorption). Comparing the presented correlations, we can also notice lower values of standard deviations (SDs) in the case of 4-CPA/RIB and 3-CPP/RIB than for the 2,4-D/RIB system, which confirms the observed smaller spread of experimental points on the corresponding adsorption isotherms. In turn, for the 4-NA/RIB system, small standard deviations of the equilibrium concentration determination on the initial concentration function and significant standard deviations of the absorbance determination on the initial concentration function can be observed. The latter undoubtedly results from the specificity of this compound. 4-NA is the only substance among those tested that has, in addition to the aromatic ring, a nitro chromophore group, which determines its high intensity of light absorption in the UV/Vis range and, therefore, a strong signal of the measured quantity (absorbance). Typically, in spectrophotometric measurements without dilution, concentrations can be determined in the range of 0.1% of the concentration value corresponding to absorbance at level 2 (i.e., in the absorbance (A) range of 0.002–2). However, in the case of 4-NA, a significant number of equilibrium solutions required dilutions, contributing to increased measurement uncertainties. Analysis of the negative impact of dilutions suggests the need to avoid them, for example, by using cuvettes of reduced thickness. Of course, this involves the need to use non-linear calibration, based on the molecular model of the phenomenon (ionization, association, influence of other components of the solution, etc.). Since non-linear calibration has its problems (which can be avoided by using large dilutions), a comparison of both methods of measuring high concentrations must be carried out, both in terms of repeatability and the possibility of systematic error.

It should be emphasized that in studies of adsorption processes, due to the indirect method of measuring the amount of the adsorbed substance using only the measurement of the equilibrium concentration of the adsorbate in the bulk phase, we are dealing with error propagation. This means that the uncertainty in the determination of the equilibrium concentration is also transferred to the determination of the amount of adsorbed substance.

If using the analysis presented above (Figure 9), we assume that the uncertainty value is proportional to the adsorption value, it will correspond to a constant relative error. This provides a simple possibility of mean square fitting in logarithmic coordinates without having to take into account variations in uncertainty. The relatively small deviations of the data from the straight line in logarithmic coordinates found (significant deviations appear only for a few isotherms) allow the use of, among others, logarithmic (linear) form of the Freundlich isotherm, with easy access to all statistical parameters. Moreover, it should be emphasized that fitting to the Freundlich isotherm is used as a basic tool for assessing adsorption data in applied research on environmental protection issues, and the analysis presented below allows for verification of the applicability of such procedures. The linear form of the Freundlich equation, which can be derived based on the theory of adsorption in energetically heterogeneous systems, has the form:

log *a* = *m*log *c_eq_* + *m*log *K* (3)

where *a*—adsorption, *K*—adsorption equilibrium constant characterizing the location of the distribution function on the energy axis, and *m*—heterogeneity parameter taking values in the range (0–1> and describing the shape of the adsorption energy distribution function. The lower the value of the m parameter, the more heterogeneous is a given system; for *m* = 1, the system is homogeneous.

The parameters of this equation characterizing adsorption equilibria were calculated using the linear regression method. The quality of fit of the theoretical isotherm to the experimental data was assessed based on the calculated values of *SD* (standard deviation) (Equation (4)), the values of SSRd (sum of squares residual), SSRg (sum of squares regression), SST (total sum of squares), and the values of the correlation coefficients R^2^ (Equation (5)).

(4) $$SD={\left[\sum_{i=1}^{L}\frac{{\left(y_{i}-{y(x}_{i}\right))}^{2}}{\left(L-k\right)}\right]}^{\frac{1}{2}},\;y_{i}={\log a}_{i,exp}\;\;\;y(x_{i})={\log a}_{i,teor}$$

where *a_i,exp_*—experimentally determined adsorption value, *a_i,teor_*—adsorption calculated theoretically, *L*—number of experimental points, *k*—number of parameters, SSRd—the sum of squares of differences calculated for each point as the square of the difference between its actual value *y*_i_ and its estimated value *y*(*x_i_*), SSRg—difference in total sum of squares and residual sum of squares, and SST—the sum of squares of differences between actual values *y_i_* and mean value *y_av_*, (SST = SSRd + SSRg)

(5) $$1-R^{2}=\frac{\sum_{i=1}^{L}{\left(y_{i}-{y(x}_{i}\right))}^{2}}{\sum_{i=1}^{L}{\left(y_{i}-y_{av}\right)}^{2}}=\frac{\sum_{i=1}^{L}{\left(y_{i}-{y(x}_{i}\right))}^{2}}{\sum_{i=1}^{L}y_{i}^{2}-\frac{1}{L}\left(\sum_{i=1}^{L}y_{i}\right)}$$

The values of the weighted average parameters were calculated from the following equation:

(6) $$\bar{z}=\frac{\sum_{i=1}^{L}z_{i}\frac{1}{{SD}_{i}^{2}}}{\sum_{i=1}^{L}\frac{1}{{SD}_{i}^{2}}}\;,\;\;\;\;\bar{z}=\bar{m},\;\log\;\bar{K};\;\;z_{i}=m_{i},\;\log K_{i}$$

In the variance assessment SD^2^(par_av_) of weighted average parameters due to the large range of their I–IV values compared to their variances SD^2^(par) (this suggests that the actual variances are larger than those determined), a variant of the formula with a scaling factor was used, which allowed for a realistic assessment of the actual uncertainty of the average value [38]:

(7) $$SD\left(\bar{z}\right)=\sqrt{\frac{\sum_{i=1}^{L}{\left(z_{i}-\bar{z}\right)}^{2}\frac{1}{{SD}_{i}^{2}}}{\sum_{i=1}^{L}\frac{1}{{SD}_{i}^{2}}}}\;\cdot \;\frac{1}{\left(L-1\right)}$$

The values of the Freundlich equation parameters *m* and *log K* and their uncertainties, weighted averages, the values of the correlation coefficient *R*^2^ and standard deviations *SD* for the adsorption logarithm, *log a*, calculated using the linear regression method, for four independent series (I–IV) of 2,4-D, 4-CPA, 3-CPP, and 4-NA adsorption measurements on the activated carbon RIB are listed in Table 3.

**Figure 10:**
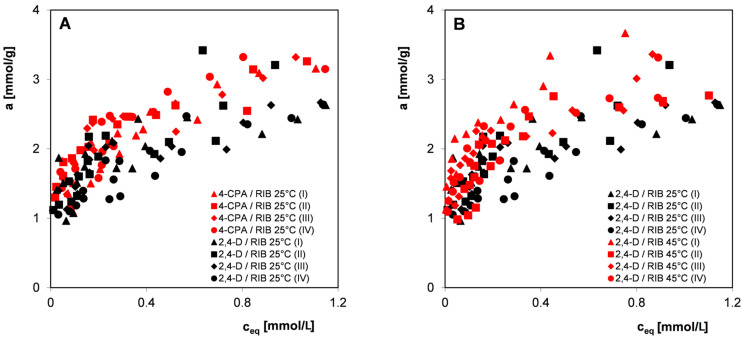

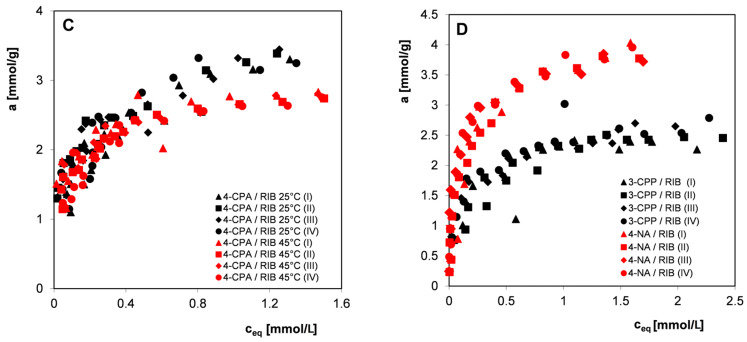
Comparison of the adsorption isotherms of 2,4-D, 4-CPA, 3-CPP, and 4-NA measured under the same (**A**,**D**) and different temperature conditions (**B**,**C**) in four measurement series.

A comparison of the values of the heterogeneity parameters m of the Freundlich equation characterizing the adsorption of 2,4-D, 4-NA, 3-CPP and 4-NA on the carbon RIB allows us to conclude that all systems are energetically heterogeneous, with the highest degree of heterogeneity demonstrated by the system with 2,4-D (the lowest m values). The adsorption equilibrium constants for 2,4-D and 4-CPA are similar, which indicates a comparable affinity of both compounds for the carbon, while for 3-CPP they are much lower. This is undoubtedly related to the experimental conditions, which determine the form of the adsorbate and the adsorption mechanism. Under acidic conditions (pH = 2), 2,4-D and 4-CPA molecules occurred mainly in their undissociated forms (pK_a_ = 3.14 and 2.81, respectively), which resulted in strong dispersion interactions between the π electrons of the adsorbate aromatic ring and the carbon graphene planes. The molecules of the above-mentioned herbicides have a chloride substituent with properties that deactivate the aromatic ring, which weakens this type of interaction with the carbon surface. The more deactivating groups, the stronger the effect determining the adsorption mechanism. The additional chloride group in the 2,4-D molecule causes this compound to have lower solubility in water than 4-CPA and has greater hydrophobic interactions with the adsorbent. On the other hand, 4-CPA has smaller geometric parameters, i.e., van der Waals volume and maximum projection area, which favors its greater packing in the pores of the adsorbent and increases adsorption (Figure 10A). Taking into account the influence of temperature conditions on adsorption, we can conclude that while for 2,4-D the adsorption increases with increasing temperature/the endothermic nature of the process (Figure 10B), for 4-CPA the effect is opposite/due to the exothermic nature of the process (Figure 10C). For both herbicides, a greater adsorption under the given temperature conditions translates into a higher value of the equilibrium constant.

The adsorption of 3-CPP was carried out from an aqueous solution without a fixed pH (~neutral), so the herbicide was in an anionic form (pK_a_ = 3.27) and interacted with the carbon surface based on electrostatic forces. The positive total surface charge of the carbon RIB under experimental conditions indicates the existence of attractive forces in the adsorbate-adsorbent system; however, the relatively low value of the zero charge point of the adsorbent (pH_pzc_ = 7.8) means that on its surface, in addition to basic groups, there are also partially dissociated acid complexes interacting with repulsive forces with the adsorbate. 4-NA is characterized by a higher adsorption equilibrium constant/affinity to the carbon surface (in comparison to 3-CPP), as a result of its neutral molecular form under measurement conditions (pK_a_ = 1), which allows interaction with the adsorbent according to the mechanism of dispersion forces. The stronger adsorption of 4-NA compared to 3-CPP (Figure 10D) may be partially related to its lower solubility in water. As in the adsorption of organic substances from the aqueous phase onto hydrophobic solids, the main driving force of the adsorption process is the hydrophobicity of the adsorbate, which is measured by its solubility. The applied fitting model (the Freundlich isotherm) does not best describe the experimental systems in a wider range of concentrations, which is particularly visible in the logarithmic scale of experimental adsorption isotherms. In the case of most systems with herbicides, the determined values of the correlation coefficient R^2^ range from 0.707 to 0.945, while the values of standard deviations SD for a log range from 0.029 to 0.071, which indicates the good quality of the experimental data. Only in the case of two systems does the correlation coefficient reach a value close to 0.5 and SD reaches as much as 0.09–0.1, which indicates that larger experimental errors were made. For systems with the nitro derivative 4-NA, the values of the correlation coefficient R^2^ are above 0.854, but the values of standard deviations SD for log a reach the value range of 0.073–0.139. The statistical test results of the determination of the Freundlich equation parameters m and log K (Table 3) reflects the presence of random errors well in individual measurement series caused by the possible influence of many variables, and usually independent factors, such as (i) the inaccurate preparation of adsorption systems; (ii) instability of external factors during the adsorption process (changes in the form of adsorbate); (iii) inaccurate dilution and measurement of adsorbate solutions; and (iv) the instability of measurement equipment. It should be emphasized that the presence of such errors is largely dictated by the inhomogeneity of the adsorbent (variation in grain size, physicochemical and structural properties of individual granules) and the method of measuring the equilibrium isotherms. The use of many independent adsorbent and adsorbate solution weights means that we are not able to prepare identical adsorption systems in individual measurement series. Moreover, the low mass of individual adsorbent samples (0.04 g) increases the probability of random errors (uncertainty inversely proportional to the square root of the sample mass). The observed scatter of individual adsorption isotherm points occurring in all four measurement series of the tested systems (Figure 6, Figure 7 and Figure 8) translates into significant uncertainties in determining the Freundlich equation parameters m and log K (SD_av_. = 7–12%). However, for most adsorption systems, one can notice much smaller uncertainties of determining the parameter m than for log K which results from a smaller error in estimating the slope of the linear relationship than the point of intersection with the ordinate axis. The analysis of the validity of the Freudlich model based on the SSRd (sum of squares residual), SST (total sum of squares), and SSRg (sum of squares regression) quantities showed that the smaller the SSRd in comparison to SST, the higher the correlation coefficient value.

### 3.3. Error Analysis in Measurements of Adsorption Isotherms from Multi-Component Systems

**Figure 11:**
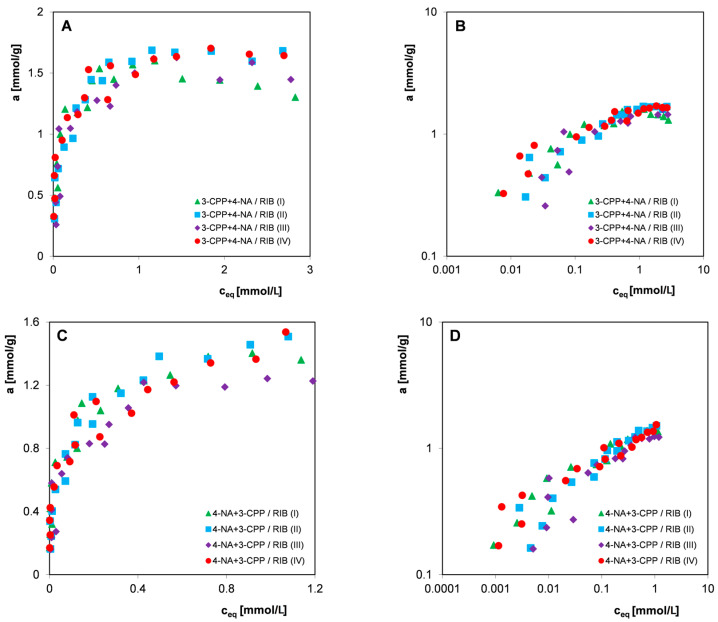
Comparison of adsorption isotherms of the pesticide 3-CPP in the presence of 4-NA (ratio of the initial concentrations of 3-CPP:4-NA was 2:1) (**A**,**B**) and 4-NA in the presence of 3-CPP (**C**,**D**) on the activated carbon RIB measured in four measurement series in linear (**A**,**C**) and logarithmic coordinates (**B**,**D**).

Figure 11 shows the adsorption isotherms of the components of the mixture 3-CPP with 4-NA (co-adsorbate) and 4-NA with 3-CPP (co-adsorbate). A high similarity of isotherms measured in four independent measurement series was observed, with a visible spread of experimental points in the area of low equilibrium concentrations. The reasons for the dispersion of experimental isotherm points, apart from the grain and structural heterogeneity of activated carbon (and, consequently, the diversity of individual carbon samples), may be the contamination of the reagents used (3-CPP and 4-NA), a larger error related to the need to dilute some of the solutions (especially for higher concentrations, where the change in concentration as a result of adsorption is small), and increased spectrophotometric measurement error that appears when measuring low concentrations, especially in the presence of other substances (apart from the basic components of the solution, signals from substances leached from the carbon appear).

**Figure 12:**
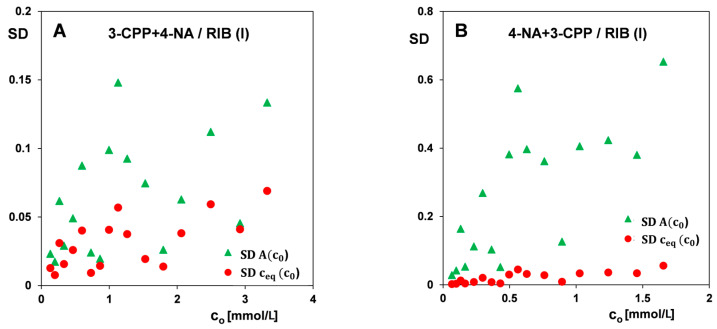
Correlations of standard deviations for the determination of equilibrium concentration and absorbance on initial concentration for the selected series of adsorption measurements of 3-CPP in the presence of 4-NA (**A**) and 4-NA in the presence of 3-CPP (**B**).

The values of standard deviation (SD_j_) for absorbance (SD_A_) and equilibrium concentrations (SD_c eq_) allow for the evaluation of the measurement error of the experimental point ‘j’ on adsorption isotherms. Figure 12 shows exemplary dependencies of the standard deviation of absorbance and equilibrium concentration determination as a function of the initial concentration for the selected measurement series of the tested adsorption system. An approximately linear trend is observed in the graphs, especially in the lower concentration range, which indicates that the error increases with rising concentration. In the case of some of the graphs, it can be stated that the relationship may be approximately a square root function or a linear function with a non-zero initial value—such a course is consistent with the model in which, apart from the dispersion proportional to the concentration (constant relative error), there is a minimal error (related the method detection limit (MDL), along with the entire analytical procedure). Moreover, in the two-component system, lower SD values for determination of the absorbance of 3-CPP than 4-NA were found, which is consistent with the observed smaller scatter of experimental points on the corresponding adsorption isotherms.

The component concentrations of complex systems were calculated upon the absorption values obtained for the two-component system and the absorption coefficients for individual substances. The basis for quantitative determinations in multi-component solutions is that all components meet the Beer–Walter law and the additivity law. It expresses the total absorbance of the system as the sum of the absorbance values of the individual components. The condition for its fulfillment by the tested system is the lack of interactions between the components absorbing radiation in the UV-Vis range. To calculate the concentrations in a binary mixture, a system of two equations with two unknown quantities was used:

*A*(*λ*_1_) = *c_eq A_ ε*_*A*1_ + *c_eq B_ ε*_*B*1_

*A*(*λ*_2_) = *c_eq A_ ε*_*A*2_ + *c_eq B_ ε*_*B*2_ (8)

where *c_eq A_*, *c_eq B_*—concentrations of substances *A* and *B* in a two-component system, *A*(*λ*_1_), *A*(*λ*_2_)—absorbances determined at wavelengths *λ*_1_ and *λ*_2_ for spectra measured for a two-component system, and *ε_A_*_1_, *ε_A_*_2_—absorption coefficients of the substance *A* at wavelengths *λ*_1_ and *λ*_2_ determined for a one-component system.

**Figure 13:**
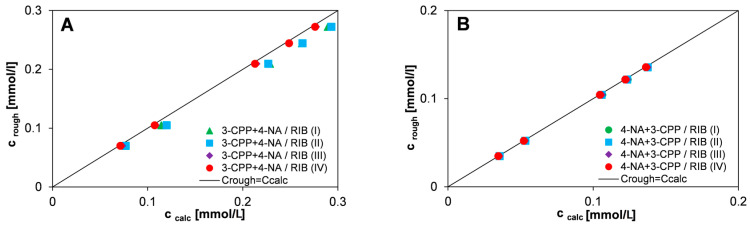
Comparison of rough concentrations of 3-CPP (**A**) and 4-NA (**B**) with concentrations calculated theoretically based on the additivity law for four series of standard two-component solutions (ratio of initial concentrations of 3-CPP:4-NA as 2:1).

For the tested two-component system, the accuracy of the quantitative determination of equilibrium concentrations based on the measured UV-Vis spectra was assessed. For this purpose, four series of standard two-component solutions containing a herbicide (3-CPP) and an accompanying substance (4-NA) with known concentrations were prepared. Absorption spectra were measured for these solutions and the concentration values of the tested substances were calculated upon the additivity law (c_calc_). The second method of determining the solution concentrations was simplified and assumed a lack of spectral interference of the spectra of both substances (c_rough_). Figure 13 compares the rough concentrations and those calculated from the additivity law for four series of standard two-component solutions.

It was observed that in the case of 4-NA, the rough concentrations (c_rough_) correspond very well to the values calculated from the additivity law (c_calc_). For 3-CPP, the error in the determination of considered quantity is slightly higher due to the partial overlap of the spectra of the pesticide and the co-adsorbate. This proves the high accuracy of the equilibrium concentration’s determination of these compounds in a complex system using the UV-Vis spectrophotometry method and calculations upon Equation (8).

**Figure 14:**
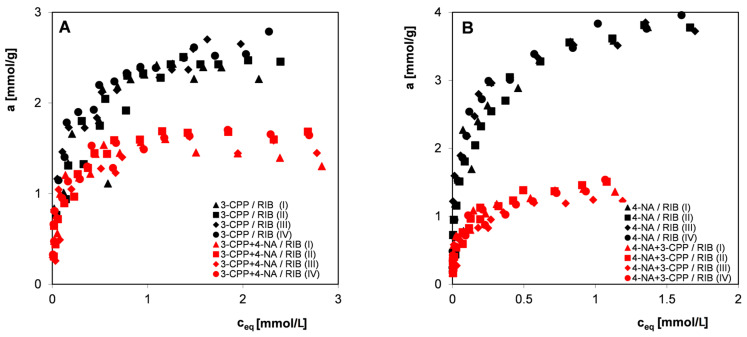
Comparison of adsorption isotherms of 3-CPP from a single-component system and in the presence of 4-NA (**A**), and a comparison of the adsorption isotherms of 4-NA from a single-component system and in the presence of 3-CPP (the initial concentration ratio of 3-CPP:4-NA is 2:1) (**B**).

Figure 14A compares the adsorption isotherms of 3-CPP from a single-component system and in the presence of 4-NA, and Figure 14B shows the adsorption isotherms of 4-NA from a single-component system and in the presence of 3-CPP.

Analyzing the herbicide adsorption from a single system and in the presence of an accompanying substance, one can observe a decrease in its adsorption, although the changes in the 4-NA adsorption in analogous conditions (herbicide as an accompanying substance) are more significant. However, differences in the initial concentration of individual components in the mixture (twice the herbicide concentration compared to the nitro derivative concentration) make it difficult to identify the substance with a greater competitive effect. Taking into account factors such as the form of adsorbates in the mixture, their solubility/hydrophobicity, the size of their molecules, and the mechanism by which adsorption takes place, it can be assumed that 4-NA should have a greater impact on herbicide adsorption than the other way around. Nevertheless, both adsorbates compete for the same adsorption centers located on the activated carbon surface. Under the experimental conditions, the adsorbent is characterized by a small surface positive charge (pH_pzc_ = 7.8), which means a possibility of the occurrence of attractive electrostatic interactions with 3-CPP anions, but weaker than with 4-aniline (dispersive interactions of π electrons of the adsorbent and aromatic rings of the adsorbate and the lone pair of electrons of the—NH_2_ moiety). 4-NA, due to its smaller molecular size, diffuses faster both in bulk solution, at the solution–solid interface, and in the pore spaces of the solid, ultimately gaining preference for adsorption in carbon micropores. Moreover, the lower solubility of 4-NA than the herbicide (greater hydrophobicity) means that, in addition to dispersion interactions, hydrophobic interactions have a greater share in its adsorption process.

## 4. Conclusions

In this work, the adsorption studies were performed for three selected herbicides, i.e., 2,4-D, 4-CPA, and 3-CPP, and the nitro derivative 4-NA in systems with the activated carbon RIB.

- The error analysis of the measurements of adsorption isotherms of single substances showed the differentiation in the values of standard deviations of the determined quantities, i.e., equilibrium concentration and absorbance. The largest deviations were obtained for the 2,4-D/RIB system, which confirmed the largest scatter of experimental points on the adsorption isotherm.
- Despite the relatively large dispersion of experimental points of adsorption isotherms, the trend in the adsorption process can be clearly determined depending on the type of adsorbate and the conditions of the adsorption process. This in turn confirms the reliability of the presented results.
- The presence of random errors and the absence of systematic and gross errors were found. The source of random errors was primarily the high inhomogeneity of the adsorbent (variation in grain sizes, physicochemical and structural properties of individual granules) and the method of measuring equilibrium isotherms consisted of the use of many independent adsorbent and adsorbate solution weights. The influence of other variables and factors such as (i) the imprecise preparation of adsorption systems; (ii) instability of external factors during the adsorption process (changes in the form of adsorbate); (iii) inaccurate dilution and measurement of adsorbate solutions; and (iv) the instability of measuring equipment is also probable.
- Significant uncertainties in determining the Freundlich equation parameters m and log K (SD_av_. = 7–12%) were found.
- The results of equilibrium adsorption studies conducted at different temperatures showed a positive trend in the dependence of adsorption on temperature for the 2,4-D/RIB system (increase in mobility of adsorbate molecules in the solution, wider penetration of the carbon porous structure), and a negative trend for the 4-CPA/RIB system (increase in solubility of the compound, increase in energy of oscillation of molecules enabling desorption).
- In acidic conditions (pH < pK_a_), the highest adsorption was obtained (mechanism–dispersive interactions between π electrons of the adsorbate aromatic ring and π electrons of the carbon graphene structure). In neutral pH conditions (pH > pK_a_), a worse adsorption efficiency was observed (mechanism–electrostatic interactions).
- Analysis of the herbicide adsorption (3-CPP) in the presence of an accompanying substance (4-NA) showed a clear decrease in comparison with adsorption from a single-component solution. The adsorption mechanism of the co-adsorbates was strongly competitive in relation to the same active sites placed on the activated carbon surface.

## Figures and Tables

**Table 1:** Physicochemical properties of the studied adsorbates [49,50,51,52].

Code	Molecular Weight [g/mol]	Ionization Constant pK_a_	Solubility [g/L]	Van der Waals Volume [Å^3^]	Maximal Projection Area [Å^2^]
4-CPA	186.59	3.14	0.96	149.71	56.50
2,4-D	221.04	2.81	0.68	163.66	60.90
3-CPP	200.62	3.27	1.20	166.83	56.64
4-NA	138.12	1.00	0.57	115.97	47.44

**Table 2:** The values of parameters characterizing the porous structure of adsorbents.

Carbon	S_BET_ ^1^ [m^2^/g]	V_t_ ^2^ [cm^3^/g]	V_mic_ (t-plot) ^3^ [cm^3^/g]	D_h_ ^4^ [nm]	D_mo_ (des. BJH) ^5^ [nm]	D_mo_ (HK) ^6^ [nm]	D_mo_ (DFT) ^7^ [nm]	pH_pzc_ ^8^
RIB	1052	0.65	0.46	2.5	3.12	0.66	0.57	7.8

^1^ S_BET_—specific BET surface area, ^2^ V_t_—total pore volume, ^3^ V_mic_—micropore volume, ^4^ D_h_—average hydraulic pore diameter, ^5^ D_mo (BJH des)_—average BJH desorption pore diameter, ^6^ D_mo_ (HK)—average micropore size from the Horvath-Kawazoe (HK) method, ^7^ D_mo_ (DFT)—average micropore size from the NLDFT, ^8^ pH_pzc_—point of zero charge.

**Table 3:** The values of Freundlich equation parameters as well as statistics for independent series (I–IV) of adsorption measurements of 2,4-D, 4-CPA, 3-CPP, and 4-NA on the activated carbon RIB.

Code	No	*m*	*log K*	SD(*log a*)	SSRg ^1^	SSRd ^2^	R^2^
2,4-D (25 °C)	I	0.178 ± 0.043	2.140 ± 0.552	0.087	0.131	0.113	0.537
	II	0.241 ± 0.034	1.841 ± 0.289	0.070	0.244	0.073	0.769
	III	0.184 ± 0.024	2.070 ± 0.298	0.054	0.166	0.044	0.792
	IV	0.286 ± 0.034	1.320 ± 0.179	0.057	0.222	0.048	0.822
	**av.**	**0.218 ± 0.025**	**1.621 ± 0.195**				
2,4-D (45 °C)	I	0.193 ± 0.021	2.698 ± 0.322	0.058	0.283	0.050	0.850
	II	0.259 ± 0.046	1.952 ± 0.223	0.071	0.270	0.075	0.783
	III	0.202 ± 0.024	2.334 ± 0.305	0.058	0.234	0.050	0.825
	IV	0.216 ± 0.019	2.204 ± 0.214	0.045	0.261	0.030	0.896
	**av.**	**0.213 ± 0.017**	**2.295 ± 0.172**				
4-CPA (25 °C)	I	0.324 ± 0.033	1.520 ± 0.168	0.052	0.261	0.040	0.866
	II	0.205 ± 0.013	2.369 ± 0.158	0.029	0.213	0.013	0.945
	III	0.223 ± 0.022	2.198 ± 0.231	0.046	0.219	0.032	0.873
	IV	0.275 ± 0.037	1.814 ± 0.263	0.065	0.236	0.064	0.786
	**av.**	**0.226 ± 0.021**	**1.989 ± 0.213**				
4-CPA (45 °C)	I	0.269 ± 0.028	1.497 ± 0.437	0.062	0.139	0.058	0.707
	II	0.210 ± 0.016	2.039 ± 0.169	0.032	0.170	0.015	0.917
	III	0.191 ± 0.021	2.242 ± 0.261	0.042	0.149	0.027	0.847
	IV	0.243 ± 0.026	1.758 ± 0.201	0.044	0.173	0.030	0.854
	**av.**	**0.227 ± 0.020**	**1.880 ± 0.203**				
3-CPP (25 °C)	I	0.253 ± 0.037	1.290 ± 0.207	0.076	0.273	0.082	0.769
	II	0.318 ± 0.026	1.049 ± 0.095	0.049	0.362	0.036	0.909
	III	0.187 ± 0.042	1.760 ± 0.426	0.101	0.196	0.152	0.563
	IV	0.256 ± 0.020	1.497 ± 0.128	0.045	0.325	0.030	0.915
	**av.**	**0.266 ± 0.022**	**1.233 ± 0.126**				
4-NA (25 °C)	I	0.356 ± 0.033	1.633 ± 0.194	0.125	1.806	0.233	0.886
	II	0.390 ± 0.041	1.504 ± 0.201	0.137	1.738	0.282	0.860
	III	0.319 ± 0.034	1.849 ± 0.251	0.139	1.702	0.291	0.854
	IV	0.314 ± 0.017	1.890 ± 0.127	0.073	1.928	0.080	0.960
	**av.**	**0.335 ± 0.021**	**1.717 ± 0.122**				

^1^ SSRd—sum of squares residual, ^2^ SSRg—sum of squares regression.

## Data Availability

The data are available from the corresponding author.
